# Air Pollution, Early Life Microbiome, and Development

**DOI:** 10.1007/s40572-018-0215-y

**Published:** 2018-09-29

**Authors:** Yvonne Vallès, M. Pilar Francino

**Affiliations:** 1 Department of Biological and Chemical Sciences, The University of the West Indies, Cave Hill campus, Cave Hill, Barbados; 2 Unitat Mixta d’Investigació en Genòmica i Salut, Fundació per al Foment de la Investigació Sanitària i Biomèdica de la Comunitat Valenciana (FISABIO-Salut Pública)/Institut de Biologia Integrativa de Sistemes (Universitat de València), Avda. Catalunya 21, 46020 València, Spain; 3 CIBER en Epidemiología y Salud Pública (CIBERESP), Madrid, Spain

**Keywords:** Gut microbiome, Infant development, Early programming, Immune disease, Obesity, Autism spectrum disorder

## Abstract

**Purpose of Review:**

We review how an altered microbiome in early life impacts on immune, metabolic, and neurological development, focusing on some of the most widespread diseases related to each of these processes, namely atopic disease, obesity, and autism.

**Recent Findings:**

The early development of the microbial communities that inhabit the human body is currently challenged by factors that range from reduced exposure to microbes, antibiotic use, and poor dietary choices to widespread environmental pollution. Recent work has highlighted some of the long-term consequences that early alterations in the establishment of these microbiotas can have for different aspects of human development and health.

**Summary:**

The long-term consequences of early microbiome alterations for human development and health are only beginning to be understood and will require in-depth investigation in the years to come. A solid understanding of how present day environmental conditions alter microbiome development, and of how an altered microbiome in early life impacts on life-long health, should inform both public health policies and the development of dietary and medical strategies to counteract early microbiota imbalances.

## Introduction

Human evolution has been punctuated by precise moments throughout history associated to key changes in lifestyle: the appearance of *Homo sapiens sapiens*, the shift from nomadic to sedentary lifestyles with the introduction of agriculture, the industrial revolution, the discovery of antibiotics, the creation of agri-business, and recently the elaboration and massive distribution of processed foods [1]. These changes appear to have had a profound effect on the evolution of human health and disease. In the last century, such effects have likely included the emergence and increased prevalence of allergies, asthma, and autoimmune diseases, with a concomitant decrease in the incidence of infectious diseases [2]. In fact, Strachan in 1989 proposed the “hygiene hypothesis”, which stated that lack of exposure to microbes during early infancy was at the source of the observed increased prevalence of allergy and asthma in westernized populations [3, 4]. Later on, the “Barker Hypothesis” (also called “Developmental Origins of Health and Disease (DOHaD)”) postulated that exposure to environmental factors during both fetus development and immediately after birth or nutritional deficiencies of the mother during gestation would result in an early programming for developing cardiovascular, neurodevelopmental, and metabolic disorders [5–8]. The latter hypotheses emphasize the notion that infancy is likely to be a critical stage in human development in which interventions could potentially prevent or decrease risk factors of latent disorders. Interestingly, there is growing evidence that early microbiome-host interactions during fetus development and early infancy are critical factors that will determine life-long health or disease states [9–11]. However, although it is clear that the first months of life represent a crucial time window in the establishment of microbiome-host interactions, the precise boundaries of this window and the impact of microbial changes during later periods of infancy and childhood on life-long disease risks remain to be determined.

Complex endeavors such as those undertaken by the Human Microbiome Project and the MetaHit Consortium have been key in highlighting the significance and complexity of the microbiota inhabiting the niches provided by the human body. It is now well accepted that the human’s gastrointestinal tract (GIT) gathers the most diverse and dense microbiota of the human body, which in turn plays fundamental roles in gut homeostasis [12, 13]. Supporting the hygiene and Barker hypotheses, there is now a great body of knowledge establishing that GIT microbiota composition during infancy and childhood are associated to an incredible array of human diseases, from GIT-related diseases (i.e., metabolic disorders such as diabetes and obesity, inflammatory bowel disease), to immune diseases and neurological disorders [14–22].

The advent of sequencing technologies that enable the deep characterization of microbial communities without the need for isolation and culturing of their individual members has revealed the great complexity of the human microbiome, as well as the presence of rare or unculturable organisms that had previously escaped detection. Interestingly, these advances have not only led to the discovery of the importance and involvement in health and disease of the human microbiome, but also to surprising breakthroughs challenging long-standing dogmas. In particular, until recently, it was believed that in health, the placenta was an impenetrable barrier to bacteria maintaining an in utero sterile environment in which the fetus developed. However, numerous recent analyses based on the amplification and high-throughput sequencing of bacterial 16S-rRNA genes have demonstrated the presence of bacteria in the placenta [23–25], umbilical cord [26], amniotic fluid [24], and meconium [27–29]. Moreover, experimental work has confirmed an efflux of bacteria from the mother’s gut to that of the fetus, as genetically labeled bacteria orally inoculated to pregnant mice are recovered from the meconium of offspring obtained by C-section [27]. Following the latter, several hypotheses have been proposed as to how bacteria can reach in health the in utero environment, including entry into the mother’s bloodstream via translocation events from the mother’s GIT and the oral cavity [23, 24, 30]. The important physiological changes occurring in the GIT during pregnancy and in particular during the third trimester entail an inflammatory state of the intestinal epithelium, similar to that present in obesity and diabetes [31], which could enhance translocation events of bacteria. In fact, in mice, enhanced bacterial translocation from the gut has been shown to take place both during late pregnancy [32] and at the early onset of type 2 diabetes [33], and, in the latter case, the dendritic cells of the immune system have been implicated in mediating the increased translocation level. The proposition that maternal bacteria reach the fetal gut is ground breaking, as these organisms could start marking the trajectories of immune, metabolic, and somatic development in utero, with enormous implications for the health of the individual [29].

**Fig. 1:**
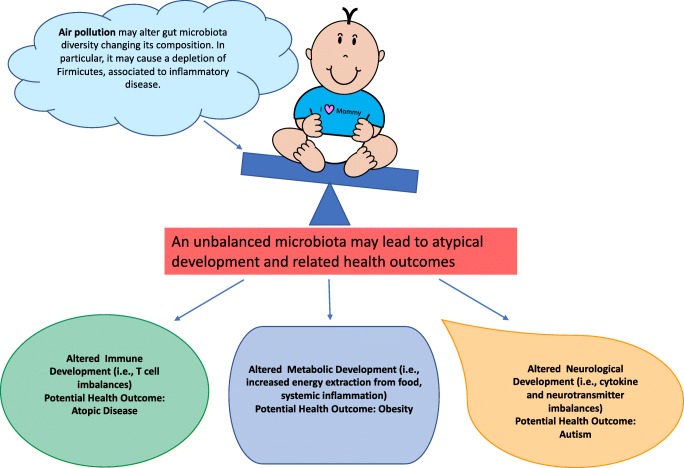
Air pollution, among many other factors, may alter the balance of the gut microbiota and contribute to altered immune, metabolic, and neurological development

Here, we review several aspects of human development and health that are known to be affected by the gut microbiome in early life, as well as the emergent evidence for air pollution as a hitherto rarely considered environmental factor that likely contributes to altering the establishment of the gut microbiome (Fig. 1).

## Air Pollution: an Environmental Factor Recently Associated to Microbiome Dysbiosis

Air pollution is the presence of harmful substances in the air that can result from natural causes (i.e., volcano eruptions, wind dust) and human activities (i.e., combustion of fuels, industry, traffic, cooking, smoking). The presence in polluted air of larger proportions of CO_2_, SO_2_, other toxic gases, chemical compounds, and different sizes of particulate matter (PM) constitutes a universal hazard to those organisms exposed to it. In fact, correlations of long-term exposures to air pollution and mortality have been addressed in several cohort-based studies in different parts of the world [34, 35]. They have demonstrated the existence of an association between long-term exposure to fine PM and an increased risk of cardiovascular and lung disease, as well as increased risk of lung cancer [36]. It has also been suggested that air pollution is associated to gastrointestinal disorders by being potentially involved in the pathophysiology of inflammatory bowel disease [37].

Air pollutants are inhaled into the lungs. The smaller particles can reach the alveolar space where they can be phagocytosed by alveolar macrophages and consequently transported to the oropharynx and into the GIT [38]. In addition, air pollutants can also enter the body through the oral cavity being directly ingested with food and liquids in significant amounts. Mutlu et al. (2011) demonstrated that exposure to PM increased the production of mitochondrial reactive oxygen species (ROS) and the release of inflammatory cytokines among other effects, increasing overall gut permeability [39]. The latter in turn can potentially affect the dynamics of the gut microbiota, possibly resulting in imbalances of this community. A recent study in which mice were exposed to ambient PM_2.5_ (PM with a diameter of 2.5 μm or less), during 8 h a day, showed that there were significant changes in the mice GIT microbial diversity and composition. Interestingly, there was a significant increase of the family S24_7 (order Bacteroidales), whose members have high host glycan degradation potential, likely being involved in the degradation of the mucus layer and therefore increasing GIT permeability [39]. Also, the proportions of Firmicutes were significantly depleted, which could be accounted for, among other reasons, by the observed disappearance of the genus *Lactobacillus*, traditionally considered as a beneficial commensal promoting GIT homeostasis [39]. This depletion of Firmicutes has been correlated in other studies to an inflammatory state of the GIT.

In 2013, Kish et al. showed that mice exposed by oral ingestion to PM_10_ from Ottawa’s urban environment, during a period of 7–14 days, presented an altered gut microbiota composition and function and exhibited an acute and chronic inflammatory response in the intestine [40]. Exposure of mice to heavy metals administered through drinking water, such as lead (10 ppm for 13 weeks [41•] or 100–500 ppm for 8 weeks [42]), arsenic (10 or 250 ppm for periods of 2, 5, and 10 weeks [43] or 3 mg L^−1^ for 90 days [44]), and iron (5 mg L^−1^ for 90 days [44]), whether independently or in a combination of arsenic and iron [44], resulted in changes of the relative abundances of taxa when comparing controls to exposed groups, as well as changes in metabolic functions. For example, in those mice exposed to arsenic and/or iron, an increased prevalence of antibiotic resistance genes was observed, implying a fitness advantage for those bacteria that possess them and increasing the potential for pathogens to acquire them as well through horizontal gene transfer.

Although human studies are still lacking, the results obtained in animal models, together with the widespread occurrence of air pollution in present day cities, raise the possibility that this environmental factor is contributing significantly to microbiome-related health issues. Beyond metals and particular matter, numerous toxic gases and other chemical compounds that can be present in polluted air could also have important effects on the gut microbiota, but, to date, their potential roles in contributing to gut dysbioses associated with modern urban life have yet to be studied. The fact that air pollution is associated to increased permeability and inflammation of the GIT [38] likely has an important impact on pregnant women exposed to it, as it could exacerbate undesirable bacterial translocation events through the gut barrier, setting the stage for an imbalance in the types of bacteria that may reach the fetal gut and seed the infant’s microbiota.

## Immune Health: Atopic Disease

The microbiota of the gut develops in close interaction with immune development in a process that can shape the main determinants of life-long propensity to immune disease. Innate immunity is most developed in the intestinal tract, where both immune and epithelial cells encode a variety of receptors for ligands of microbial origin [45, 46]. Engagement of these receptors results in the production of cytokines that will direct the differentiation of the naïve T cells of the adaptive immune system. These cells can differentiate into regulatory cells (Tregs) or into helper cells, such as Th1, Th2, and Th17 [46, 47]. The activity of Tregs results in a variety of anti-inflammatory roles and suppresses the activation and development of other naïve T cells towards Th types [47–49]. The different Th cells play specific roles in shaping the immune response [47, 50, 51] and produce cytokines that suppress other Th types [52, 53]. Thus, an aberrant microbial colonization can produce an imbalance among the different types of T cells, and the consequent immune deregulation can generate a variety of pathological outcomes, ranging from atopy to autoimmune disease [9–11, 54–58].

In support of this notion, it has long been known that a reduced exposure to microbes increases the likelihood of diseases related to immune imbalances [11]. For instance, infants that have higher numbers of siblings, that co-inhabit with household pets, that attend group day care at an earlier age, or that live in farms have a much lower incidence of atopic disease, presumably mediated by their higher exposure to microbes [4, 59–61]. Nowadays, a large number of studies have been able to demonstrate associations between gut microbiota composition during infancy and early childhood and a variety of atopic diseases [9, 10, 45, 58, 62–68, 69••]. In particular, a deficiency in bifidobacteria has often been linked to increased risks of atopy, although two large prospective studies could not confirm such an association [68, 70]. Discrepancies may arise from the fact that different human populations will have distinct genetic backgrounds and may carry different bacterial species and strains of *Bifidobacterium*. More recently, a large analysis of the gut microbiota of children enrolled in the Canadian Healthy Infant Longitudinal Development (CHILD) Study has shown that infants at risk of asthma had a lower abundance of the Firmicutes genera *Lachnospira*, *Veillonella*, *Faecalibacterium*, and *Rothia*, accompanied by decreased levels of fecal acetate and dysregulation of enterohepatic metabolites. Importantly, the inoculation of germ-free mice with these four bacteria ameliorated airway inflammation, demonstrating their causal role in preventing asthma [69••]. On the other hand, increased abundances of *Clostridium* and of the enteric bacteria have also been associated with atopic disease [58, 62, 64–67, 71–75], as well as an overall decrease in the diversity of the infants’ GIT microbiota [62, 67]. Moreover, a study of meconium samples from the INMA cohort has shown that the association of low diversity and high levels of enteric bacteria with atopic disease may be initiated by maternal factors in utero [28]. In this work, these compositional patterns were already detected in the meconium of children who developed eczema by 4 years of age or whose mothers had a history of eczema. Finally, the fecal levels of bacterial fermentation products, such as short-chain fatty acids, have also been associated with food allergies [76, 77], confirming that the metabolic output of the microbiota is relevant for atopy development.

## Metabolic Disorders: Obesity

Obesity, or excessive body fat accumulation, is a complex disease characterized by a low-grade systemic inflammatory tone that is influenced by genetic, environmental, and lifestyle factors. Obesity has become in the past few decades one of the major public health concerns worldwide, as its prevalence has increased at an alarming rate in adults and what is more disturbing, in children, particularly in urban settings of low- and middle-income countries [78]. In fact, the World Health Organization (WHO) reported that the number of overweight children under the age of 5 was estimated in 2013 to be over 42 million worldwide [78]. These facts are unsettling as overweight/obese children are likely to remain overweight/obese when adults and are more prone to develop non-communicable diseases like diabetes and cardiovascular disorders at a younger age [78, 79].

One of the main functions of the GIT microbiota is the extraction of energy from otherwise indigestible dietary polysaccharides, which can be used or stored in adipocytes [80, 81]. In fact, Bäckhed et al. in 2004 were able to show that germ-free (GF) mice accumulated less fat than wild-type mice and that introducing gut microbiota into the GF mice resulted in an increase of body fat accumulation despite a low-calorie intake diet [82].

The GIT’s microbiome in healthy individuals is characterized by a highly diverse taxonomic composition where most of the organisms pertain to five major phyla: Firmicutes, Bacteroidetes, Actinobacteria, Proteobacteria, and Fusobacteria [83]. While obesity has been linked in multiple studies comparing lean and obese individuals with changes in the abundance ratio between Firmicutes and Bacteroidetes, conflicting results have been reported as well [80, 81, 84–86]. What is well established is that obesity is related to a decrease in microbial diversity in general and that this phenotype predisposes the individuals to further inflammation [87].

The prevalence of obesity among women in age of reproduction worldwide has considerably increased in the past few decades and with it the predisposition of their infants to also develop obesity during childhood [88, 89]. The latter can be due to inheritance of obesity susceptibility genes and/or exposure to high-calorie diets but also, as noted above, to the presence of an aberrant GIT microbiota. Since initial colonization of the infant’s GIT starts in utero and may involve bacteria deriving from the mother’s GIT, we expect that the infant’s GIT will be influenced by the mother’s condition, presenting from birth an anomalous microbial community.

Multiple studies have addressed this question. Among them, Collado et al. (2010) looked at the GIT’s microbiota composition in infants at 1 and 6 months of age, finding that at 6 months, there was a correlation between microbiota composition and the obesity status of the mother [90]. In contrast, Laursen and colleagues (2016) found no association between the mother’s body mass index (BMI) and the infant’s gut microbiota [91]. Stanislawski et al. (2017) explored whether pre-pregnancy overweight/obesity and gestational weight gain were associated to different gut microbial communities at the time of delivery as well as with the infants’ gut microbiotas. They found that although the maternal gut microbiota composition was associated to their overweight/obese and gestational weight gain status, there was only a weak association to their infants’ gut microbiota composition [92]. Yet, Cerdó et al. (2018) found that the mother’s pre-pregnancy BMI status was actually associated to the functional profile of the infant’s microbial community, suggesting a possible role of maternal imprinting in the selection of gut microbial communities with specific functional potentials [16]. In spite of the inconclusive results observed in the latter studies, it is important to remember that the initial stages of an infant’s gut microbiota establishment are hectic and dramatic changes can occur in short lapses of time, thus complicating the possibility of finding reliable associations [93]. Moreover, many additional variables can confound association analyses due to their effects on the infant’s microbiota composition, such as the mode of birth, milk supply (breastfeeding versus formula), solid food introduction, exposure to antibiotics (strength, duration, and number of doses), and exposure to the surrounding environment [15, 93–95, 96•, 97, 98].

On the other hand, the relevance of early life gut microbiota for the development of obesity has been clearly demonstrated. Several analyses have shown that infants with a higher abundance of *Bifidobacterium* during the first year of life have lower adiposity levels, BMI, and obesity risk at later ages, ranging from 18 months to 7 years [96•, 99, 100]. These studies demonstrate that dysbiosis of the GIT’s microbiota does precede the onset of obesity. Interestingly, one of these studies revealed that the influence exerted by the abundance of *Bifidobacterium* and other taxa on BMI appeared to be especially strong among children with a history of antibiotic use [99].

## Neurological Disorders: Autism

Autism spectrum disorders (ASD) are an array of neurodevelopmental disorders characterized mainly by deficiencies in social behavior and communication skills, the prevalence of which has dramatically risen in the past few decades [101]. Initially believed to be a consequence only of environmental exposures, there is now enough evidence that a strong neurodevelopmental component is also at play [101]. Brain development in mammals starts early in utero and continues after birth, being continuously influenced by cue signals from the environment. The latter can have, later on, a profound impact on brain and behavior development during early childhood, in line with the “Barker’s hypothesis” [6]. It is well accepted that normal development of the fetus brain while in utero requires a specific balance of cytokines in both the maternal and fetal environments [102].

Remarkably, an association has been found between ASD and the prevalence of gastrointestinal disorders [103]. In addition, recent work has demonstrated that the composition and diversity of the gut microbiome, which, as discussed above, plays a key role in the modulation of immune system responses (i.e., cytokine and neurotransmitter secretion), are significantly associated with cognition and neurological disorders such as ASD in human infants and children [20, 104–106]. In fact, epidemiological studies and experimental work with mice have revealed a direct link between microbial pathogen infections during the prenatal phase and post-natal development of autism and behavioral abnormalities, respectively [7, 107]. Moreover, Diaz Heijtz et al. (2011) found that GF mice displayed higher motor activity and less anxiety when compared to mice with a normal gut microbiota (SPF) and showed that GF mice inoculated early on with SPF microbiota displayed motor abilities and anxiety levels similar to normal SPF mice. Although only males were used in this study, the integration of measurements of motor activity, anxiety-like behavior, neurochemical analysis, and gene expression, among others, strengthens the results observed, linking the GIT microbiota to the gut-brain axis [107].

Furthermore, based on the fact that epidemiological studies have demonstrated the association between maternal infection and increased autism risk in the offspring, Hsiao et al. (2013) showed that injecting pregnant mice of the Maternal Immune Activation (MIA) model with the viral mimic polyinosinic/polycytidylic acid (poly(I/C)), a synthetic double-stranded immune-stimulant, resulted in offspring exhibiting characteristic symptoms of ASD (i.e., communicative and social impairments) and defects of intestinal barrier integrity [108]. In addition, MIA offspring presented a similar microbial composition to that observed in humans affected by ASD which was significantly different from offspring controls, with differences driven mostly by changes in the diversity of members of the Clostridia and Bacteroidia classes. More importantly, treatment of the MIA offspring with inoculations of *Bacteroides fragilis* corrected intestinal barrier integrity and attenuated the abnormal communicative and social behaviors observed [108]. These findings and the fact that ASD shares many symptoms with many other neuropathophysiological disorders are encouraging as they show the potential of developing therapies by modulation of the gut microbiome as a safe and effective way of treatment.

More recently, Kang et al. (2017) demonstrated that microbiota transfer therapy (MTT) with a standardized human gut microbiota, after 14 days of vancomycin treatment followed by 12–24-h fasting with bowel cleansing, was able to alter the gut microbiome and virome of children with ASD and to improve GIT and behavioral symptoms, whether administered orally or rectally [109••]. More importantly, the improvements lasted for 8 weeks after the end of treatment, suggesting a long-term impact, and indicating that MTT could be a promising approach to treat ASD. Despite these promising results, it is important to take into account that the number of participants included in the study was low and that patients were not necessarily homogeneous in the GI symptoms that they presented. In addition, the use of placebo controls would have strengthened the results obtained.

Environmental factors like air pollution might trigger or exacerbate ASD by impacting the gut microbiota, which in dysbiosis potentially leads to an increased gut barrier permeability, increasing in turn bacterial translocation events and potential leakage of other pathogens and compounds (antigens and bacterial metabolites) that can induce inflammatory responses and indirectly impinge on brain functions.

## Conclusions

In the upcoming years, further efforts should focus on delineating the variety of long-term health outcomes that can result from or be aggravated by an unbalanced microbiota development. Because we now know that there are fetal microbial GIT communities, further research should better define how and when such communities are formed and to which extent they are capable of influencing human development and disease risk during gestation. In this respect, it is also crucial to invest research efforts in understanding the health and environmental factors that shape the maternal microbiome during pregnancy and how these affect the types of bacteria that reach the fetus. Regarding timing and mode of birth, although it is well established that these factors have a strong effect on early microbiome development, much research remains to be done in order to elucidate the specific mechanisms through which these early events impinge on the establishment of microbiome-host interactions, at the immune, metabolic, and neurodevelopmental level. Further, it will also be necessary to delineate the post-natal time window during which the main traits of such microbiome-host interactions are defined, as this will be the critical period in which any preventative or therapeutic interventions aimed at modulating the microbiome should be most effective. In addition, it will be important to investigate whether this time window is altered by the many variables that affect the course and pace of microbiome development during the first months of life, such as type of milk feeding, solid food introduction, exposure to antibiotics, and the many environmental and lifestyle factors that shape infant exposure to microbes [15, 93–98].

Beyond infancy, the extent to which microbiome composition may be altered by different factors during childhood and adolescence, and the subsequent health effects, have received to date little attention. On the other hand, microbiotas other than the one present in the gut have been comparatively neglected in terms of understanding their development and the potential effects of early life alterations on later health and should be further investigated in this respect. Ideally, the establishment of lasting longitudinal birth cohorts should be promoted, so that the evaluation of microbiome-related outcomes can be extended into adulthood. Such long-term projects should aim at gathering a wide scope of medical and environmental metadata, as well as samples enabling the study of bacterial communities in a variety of body sites, including, beyond the GIT, the oral cavity, respiratory system, skin, and urogenital tract. In particular, it is clear that environmental factors such as air pollution and exposure to chemical contaminants present in food and water have received little attention in spite of their strong potential to impinge on microbiome development and associated health outcomes.

On the positive side, the same malleability that renders early microbiome development susceptible to negative alterations should make it responsive to strategic interventions aimed at modulating the microbiome to promote health. Research on early microbiome development and on the effects of present day life conditions on this critical process will enable novel approaches in public health, nutrition, and medicine that ensure the establishment of a health-promoting microbiota. Such approaches will likely include the development of preventive or therapeutic treatments based on the early administration of beneficial bacteria and of nutritional supplements capable of promoting their growth.
